# A simple gene set-based method accurately predicts the synergy of drug pairs

**DOI:** 10.1186/s12918-016-0310-3

**Published:** 2016-08-26

**Authors:** Yu-Ching Hsu, Yu-Chiao Chiu, Yidong Chen, Tzu-Hung Hsiao, Eric Y. Chuang

**Affiliations:** 1 Graduate Institute of Biomedical Electronics and Bioinformatics, National Taiwan University, Taipei, Taiwan; 2 Greehey Children’s Cancer Research Institute, University of Texas Health Science Center at San Antonio, San Antonio, Texas USA; 3 Department of Epidemiology and Biostatistics, University of Texas Health Science Center at San Antonio, San Antonio, Texas USA; 4 Department of Medical Research, Taichung Veterans General Hospital, Taichung, Taiwan; 5 Bioinformatics and Biostatistics Core, Center of Genomic Medicine, National Taiwan University, Taipei, Taiwan

**Keywords:** Synergy prediction, Drug combination, Gene set enrichment analysis

## Abstract

**Background:**

The advance in targeted therapy has greatly increased the effectiveness of clinical cancer therapy and reduced the cytotoxicity of treatments to normal cells. However, patients still suffer from cancer relapse due to the occurrence of drug resistance. It is of great need to explore potential combinatorial drug therapy since individual drug alone may not be sufficient to inhibit continuous activation of cancer-addicted genes or pathways. The DREAM challenge has confirmed the potentiality of computational methods for predicting synergistic drug combinations, while the prediction accuracy can be further improved.

**Methods:**

Based on previous reports, we hypothesized the similarity in biological functions or genes perturbed by two drugs can determine their synergistic effects. To test the feasibility of the hypothesis, we proposed three scoring systems: co-gene score, co-GS score, and co-gene/GS score, measuring the similarities in genes with significant expressional changes, enriched gene sets, and significantly changed genes within an enriched gene sets between a pair of drugs, respectively. Performances of these scoring systems were evaluated by the probabilistic c-index (PC-index) devised by the DREAM consortium. We also applied the proposed method to the Connectivity Map dataset to explore more potential synergistic drug combinations.

**Results:**

Using a gold standard derived by the DREAM consortium, we confirmed the prediction power of the three scoring systems (all *P*-values < 0.05). The co-gene/GS score achieved the best prediction of drug synergy (PC-index = 0.663, *P*-value < 0.0001), outperforming all methods proposed during DREAM challenge. Furthermore, a binary classification test showed that co-gene/GS scoring was highly accurate and specific. Since our method is constructed on a gene set-based analysis, in addition to synergy prediction, it provides insights into the functional relevance of drug combinations and the underlying mechanisms by which drugs achieve synergy.

**Conclusions:**

Here we proposed a novel and simple method to predict and investigate drug synergy, and validated its efficacy to accurately predict synergistic drug combinations and to comprehensively explore their underlying mechanisms. The method is widely applicable to expression profiles of other drug treatments and is expected to accelerate the realization of precision cancer treatment.

**Electronic supplementary material:**

The online version of this article (doi:10.1186/s12918-016-0310-3) contains supplementary material, which is available to authorized users.

## Background

Development of effective treatments for cancers is an essential issue in clinical therapeutics. Thanks to the accumulated knowledge of cancer pathology, cancer treatments have been gradually shifting from the one-size-fits-all cytotoxic approach to the precision medicine that targets specific pathological features on a personalized basis [1]. Based on the reductionist “one gene-one disease” premise [2], antitumor drugs are designed to inhibit the growth of cancer cells by targeting essential genes or pathways with high specificity and efficacy, reducing damages to the normal cells. However, even with such advance in cancer therapeutics, some patients still suffer from refractory responses due to the development of drug resistance. Because of the complexity and heterogeneity of cancers, single drug alone may not be effective enough to completely and continuously suppress the activity of critical oncogenes or pathways. A common feature of drug resistance is a continuous activation of drug targets or their downstream signaling pathways [3]. On the other hand, combinatorial drug therapy may effectively circumvent the acquisition of drug resistance and optimize the efficacy of anticancer drugs.

Response to combinatorial drug therapy is optimized when a drug combination achieves greater (synergistic) effects than independent effects [1]. The synergy of drugs can be assayed by testing the inhibition of tumor cell growth by individual drugs and their combinations in vitro, followed by a mathematical formulation by Loewe additivity or Bliss independence [1, 2]. However, given the large number of drugs that are approved by FDA or under clinical trials, it seems to be impractical to experimentally test the synergy of all possible drug combinations, motivating the development of efficient computational methods for systematic screening and prediction of synergistic combinations.

Previous studies have proposed a handful of computational approaches to analyze high-throughput molecular datasets for predicting the synergy of drug combinations [4–6]. One of the computational approaches is devised based on the gene expression profiles achieved from treatments of individual drugs ([7–9]; reviewed in [2]). With the accumulation of gene expression profiles of drug treatments [10–12], the performance of such approach to model the underlying mechanisms of drug treatment can be improved. In a recent community computational challenge, namely the DREAM challenge (http://dreamchallenges.org/andhttps://www.synapse.org/#!Synapse:syn2785778/wiki/70252), 31 computational methods were developed to predict the synergistic effects of a total of 91 drug pairs by using gene expression profiles and evaluated against an experimental gold standard [13]. The promising results achieved by these methods highlight that *in silico* synergy prediction is possible and may greatly reduce the costs to screen for synergistic drug combinations. However, the accuracy of predictions still remains to be optimized [13].

The proposed study is motivated by the need for an improved prediction algorithm to systematically screen for synergistic drug combinations based on whole-genome expression profiles. According to the observations of previous in vitro studies, two synergistic drugs may target common signaling pathways to reinforce their individual effects [14, 15]. We hypothesized that drugs with synergistic effects perturb similar biological functions, or similar genes in a biological function in cells. To test the hypothesis, we designed three prediction scores based on the similarities in gene and/or functional level changes induced by two individual drug treatments. Here the functional changes were modeled by a gene set enrichment analysis, which summarizes expressional profiles to the functional level and uncovers systematic, even when subtle, changes in biological functions [16]. Specifically, we identified commonly enriched gene sets, measured by gene-set enrichment scores, between two drug treatments. Degree of overlapping of these gene sets and of the disturbed gene components in these gene sets between two drugs were used to construct three scoring systems. Performance of the scores was tested against the gold standard of 91 drug pairs provided by the DREAM challenge. In order to identify potential synergistic combinations over a broader range of drugs, we applied our method to the Connectivity Map (CMap) dataset [10], which includes the expression profiles associated with more than a thousand compounds, and identified both previously confirmed and novel synergistic drug pairs for breast cancer. Furthermore, since the gene sets represent biological functions, our prediction models also provide biological insights into the underlying mechanisms regulated by synergistic drugs.

## Methods

### Datasets from the DREAM challenge

We downloaded the expression profiles of Diffuse Large B-Cell Lymphoma OCI-LY3 cell line treated independently with 14 drugs at 2 different concentrations or DMSO (the control) at 6, 12, or 24 h after treatment from the Gene Expression Omnibus (accession ID GSE51068). The platform used to profile the gene expression was Affymetrix Human Genome U219 Array. We used the expression profiles obtained at 24 h because the IC_20_ values were measured only at 24 and 48 h in the original study [13].

A gold standard ranking of the 91 drug combinations was provided by the DREAM consortium to evaluate the performance of prediction methods. The drug combinations were ranked according to the value of excess over Bliss (EOB), the difference between the expected fractional inhibition and the induced fractional inhibition by drug combination [13]. A pair of drugs with EOB ~0 have additive effect while positive (or negative) EOB values represent synergistic (or antagonistic) effects.

### CMap dataset

A total of 2,588 expression profiles of a breast cancer cell line, MCF7, treated by 1,118 small-molecule perturbagens were downloaded from the CMap database [10].

### Gene sets

Gene sets were downloaded from the BioMart website of Ensembl (http://www.ensembl.org/index.html) and the Molecular Signature database (MSigDB v3.1, http://www.broadinstitute.org/gsea/msigdb/index.jsp) [16]. Totally 5,357 gene sets of Gene Ontology biological process, molecular function, and cellular component, chemical and genetic perturbations, and oncogenic signatures were collected. To eliminate gene sets with highly overlapped gene components, we clustered these gene sets by kappa statistics and selected one gene set from each cluster as a representative gene set. A total of 2,181 representative gene sets were selected for subsequent analysis.

### Gene set enrichment analysis

We started the analysis by performing gene set enrichment analysis for each drug in order to investigate the functions regulated by the drug. The enrichment score was calculated as

$$ {S}_l = \frac{1}{N^l}{\displaystyle \sum_{j=1}^{N^l}}{Z}_{i,j} $$

where ${N}^l$ is the number of genes in gene set *l*, and ${Z}_{i,j}$ is the delta *z*-score of gene *j* in sample *i*. The delta-*z* score was achieved by *z*-transforming the expression value of gene *j* in all samples (${X}_{i,j}$) followed by a subtraction of the *z*-transformed DMSO-treated normal control, as the equation below

$$ {Z}_{i,j} = \frac{X_{i,j} - {\mu}_j}{\sigma_j} - \frac{X_{DMSO,j}-{\mu}_j}{\sigma_j} $$

where ${\mu}_j$ stands for the mean of the gene *j* and ${\sigma}_j$ is the standard deviation of the gene *j* among all samples. In order to evaluate the statistical significance of an enrichment score, we permuted the dataset for 1,000 times and calculated a *P*-value from the empirical distribution. Benjamini-Hochberg adjustment was performed to avoid multiple testing problems [17]. Adjusted *P*-value < 0.05 was set as a threshold to define significantly enriched gene sets. Non-informative gene sets, defined as those falling within the *L*_*0.05*_ range

$$ {L}_{0.05}=\pm 1.96\frac{1}{\sqrt{N}} $$

[18], where *N* is the number of genes in the gene set, were eliminated from subsequent analysis.

### Prediction scores

Our hypothesis is that the synergy of two drugs (say, *d*_*1*_ and *d*_*2*_) can be achieved by modulating common genes, similar biological functions, or common gene components within these biological functions. Accordingly, we devised three scoring methods. Suppose there are *n* commonly enriched gene sets between the two drugs. The first score (co-gene score) measures the co-disturbed genes between two drug treatments. The co-gene score for a pair of drugs *d*_*1*_ and *d*_*2*_ is

$$ co- gene\  scor{e}_{d_1,{d}_2}=\frac{g_{d_1,{d}_2}}{G} $$

where $G$ and ${g}_{d_1,{d}_2}$ are the total number of genes and the number of commonly regulated genes with significant expressional changes (with *P*-value for delta *z*-scores < 0.05) between *d*_*1*_ and *d*_*2*_. The second score (co-GS score) is constructed based on the similarity in enriched gene sets between the two drugs. Mathematical definition of the co-GS score is given by

$$ co-GS\  scor{e}_{d_1,{d}_2}=\frac{n_{d_{1,}{d}_2}}{L} $$

with $L$ representing the total number of gene sets, and ${n}_{d_{1,}{d}_2}$ denoting the number of gene sets with significant enrichment in both drugs. The third score, namely the co-gene/GS score, is an average percentage of commonly disturbed genes within the co-enriched gene sets:

$$ co- gene/GS\  scor{e}_{d_1,{d}_2}=\frac{{\displaystyle {\sum}_l}\frac{N_{d_1,{d}_2}^l}{N^l}}{n_{d_{1,}{d}_2}} $$

where ${N}_{d_1,{d}_2}^l$ denotes the number of genes with significantly changes (with *P*-value for delta *z*-scores < 0.05) in both drug treatments within a co-enriched gene set *l* for *d*_*1*_ and *d*_*2*_.

### Performance evaluation

To evaluate the performance of the proposed prediction scores, we employed the probabilistic c-index (PC-index) developed by the DREAM consortium [13], which was modified from the concordance index (c-index). The c-index computes the proportion of concordance between predicted and experimentally validated ranks by defining a score ${t}_{q,r}$ for quantifying the concordance for two pairs of drugs *q* and *r* ($q,r\in \left[1,91\right]$). The score is given by

$$ {t}_{q,r} = \left\{\begin{array}{c}\hfill 0,\  if\left({u}_q>{u}_r\ \&\ {v}_q < {v}_r\  or\ {u}_q<{u}_r\ \&\ {v}_q > {v}_r\right)\hfill \\ {}\hfill 1, if\left({u}_q>{u}_r\ \&\ {v}_q > {v}_r\  or\ {u}_q<{u}_r\ \&\ {v}_q < {v}_r\ \right)\hfill \end{array}\right. $$

and the c-index is the average of all ${t}_{q,r}$ given $q\ne r$

$$ c- index = \frac{{\displaystyle {\sum}_{\begin{array}{c}\hfill q=1,\dots, 90\hfill \\ {}\hfill r=q+1,\dots, 91\hfill \end{array}}}{t}_{q,r}}{\left(\begin{array}{c}\hfill 91\hfill \\ {}\hfill 2\hfill \end{array}\right)} $$

where *u* stands for the experimentally validated ranks and *v* is the predicted ranks. Considering the noise introduced during the experiment processes and high-throughout profiling, the DREAM consortium modified the scoring function by introducing an error function (*erf*) and computed the PC-index as follows

$$ t{\hbox{'}}_{q,r} = \left\{\begin{array}{c}\hfill \frac{1}{2}+\frac{1}{2}erf\left(\frac{EO{B}_q-EO{B}_r}{\sqrt{se{m_{EO{B}_q}}^2+se{m_{EO{B}_r}}^2}}\right),\  if\ {v}_q < {v}_r\hfill \\ {}\hfill \frac{1}{2}-\frac{1}{2}erf\left(\frac{EO{B}_q-EO{B}_r}{\sqrt{se{m_{EO{B}_q}}^2+se{m_{EO{B}_r}}^2}}\right), if\ {v}_q > {v}_r\hfill \end{array}\right. $$

and

$$ PC- index = \frac{{\displaystyle {\sum}_{\begin{array}{c}\hfill q=1,\dots, 90\hfill \\ {}\hfill r=q+1,\dots, 91\hfill \end{array}}}t{\hbox{'}}_{q,r}}{\left(\begin{array}{c}\hfill 91\hfill \\ {}\hfill 2\hfill \end{array}\right)} $$

where ${EOB}_q$ is the excess over Bliss of a drug pair *q*, provided in the gold standard profile, and ${sem}_{EOB_q}$ is the standard error of the mean of ${EOB}_q$. When there is a concordance between predicted and experimentally validated ranks, the score $t{\prime}_{q,r}$ falls between 0.5 and 1; otherwise, $t{\prime}_{q,r}$ will be within [0,0.5]. The DREAM consortium tested the range of the PC-index in the gold-standard dataset and found that the maximum PC-index (PC_max_) was 0.90 and the minimum PC-index (PC_min_) was 0.10; the normalized PC-index is defined as

$$ PC- inde{x}_{norm} = \frac{PC- inde x - P{C}_{min}}{P{C}_{max} - P{C}_{min}}. $$

For the scoring method with the highest PC-index, we computed the area under ROC curve (AUC) and performed the precision analyses as described by Bansal et al. [13]. To calculate AUC, first, drug pairs with synergy and antagonism were defined from the gold standard profile. These drug pairs were identified by the DREAM consortium using the signal to noise ratio (SNR) computed as

$$ SNR = \frac{mean\kern0.5em EOB}{sem\kern0.5em  of\ EOB} $$

where *sem* is the standard error of the mean of the EOB. If the SNR of a drug pair is greater than 2 and its EOB is positive, it is defined as a synergistic drug pair. Oppositely, if the SNR of a drug pair is greater than 2 and its EOB is negative, it is defined as an antagonistic drug pair. Other drug pairs were defined as additive. In the original paper [13], sixteen drug pairs in the gold standard were defined as synergistic, 36 drug pairs as antagonistic, and 39 drug pairs as additive. We then ranked all drug pairs according to the co-gene/GS score in a decreasing manner. The true positive rate (${TPR}_i$) and false positive rate (${FPR}_i$) for synergy prediction were calculated as

$$ TP{R}_i = \frac{T{P}_i}{T{P}_i+F{N}_i} $$

$$ FP{R}_i = \frac{F{P}_i}{F{P}_i+T{N}_i} $$

where ${TP}_i$, $FN$, ${FP}_i$, and ${TN}_i$ denote the numbers of true positives, false negatives, false positives, and true negatives, respectively, given the top *i* drug pairs were called as synergistic. The ${TPR}_i$ and ${FPR}_i$ for antagonism predictions were calculated similarly, while the drug pairs were ranked based on the prediction scores in an increasing order. The results were visualized using ROC curves and corresponding AUC values were computed. Furthermore, we calculated the precision for synergy and antagonism prediction as

$$ Precision(synergy) = \frac{T{P}_{16}}{16} $$

$$ Precision(antagonism) = \frac{TP{\hbox{'}}_{36}}{36} $$

where $TP{\prime}_{36}$ is the number of true positives when the top 36 predicted drug pairs in the increasing order were called as antagonistic.

## Results

### Model overview

**Fig. 1:**
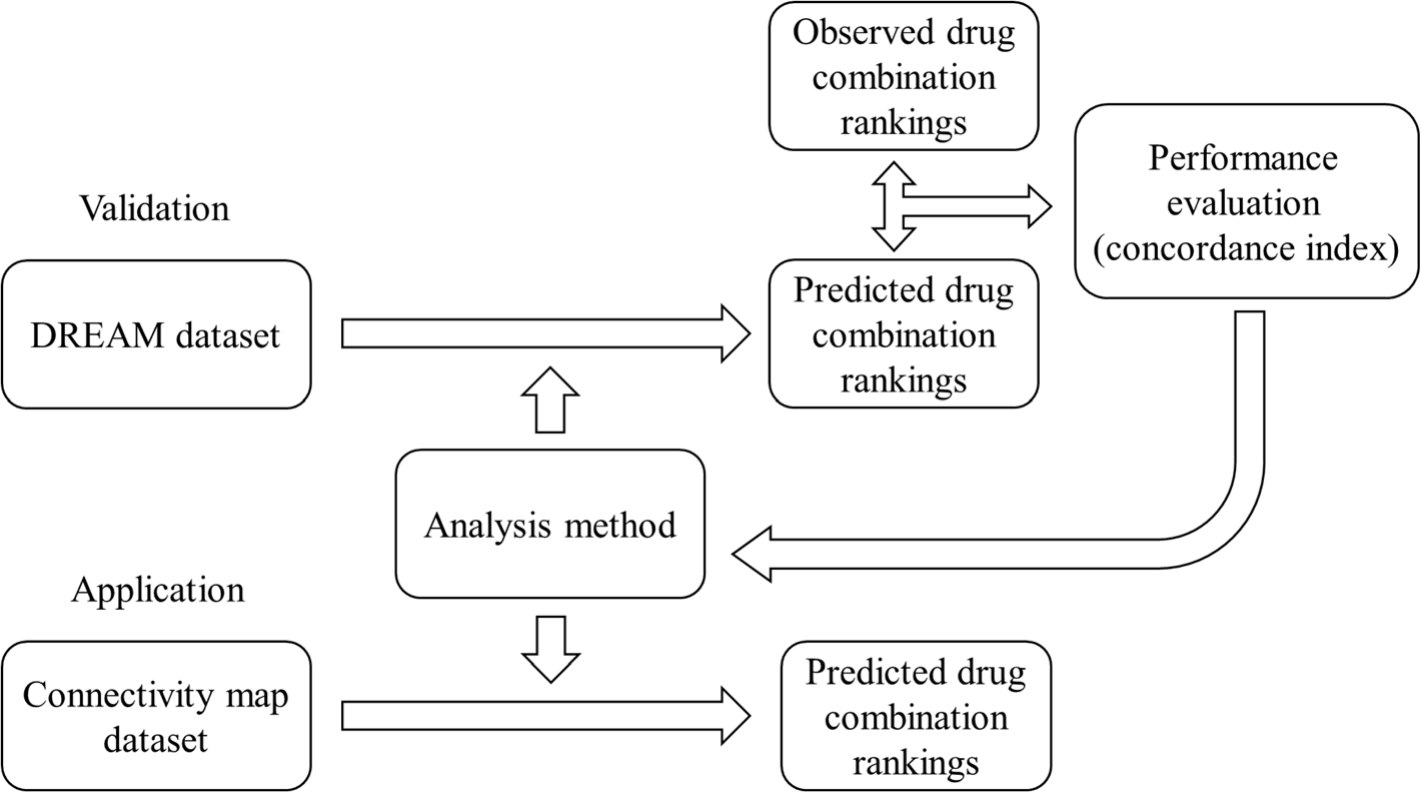
Overall design of this study. The study was aimed to test the hypothesis that synergy of two drugs can be determined by regulating a common pool of functions and/or genes. Addressing the hypothesis, three prediction methods were devised. We used the DREAM gold standard dataset to validate the methods. After confirming the hypothesis, we then employed the best-performing method to investigate synergistic effects over a wider collection of drugs using the Connectivity Map (CMap) dataset

In the present study we aim to test the hypothesis that similarities in expressional/functional changes induced by two drug treatments can predict the synergy of drugs. Figure 1 shows the overall design of this study. Based on the hypothesis, we devised three prediction scores. We tested performance of these scores by an experimentally assessed gold standard provided by the DREAM consortium, which was composed of a synergy ranking of 91 pairwise combinations among 14 well-known drugs. The rank of synergistic effects of the 91 combinations predicted by each score was compared to the gold standard and the concordance of the ranks was measured by the PC-index (detailed in the Methods). In order to extend the scale of our prediction, we applied the best performing scoring method to the CMap dataset that comprises expressional profiles of more than 1,000 compounds.

**Fig. 2:**
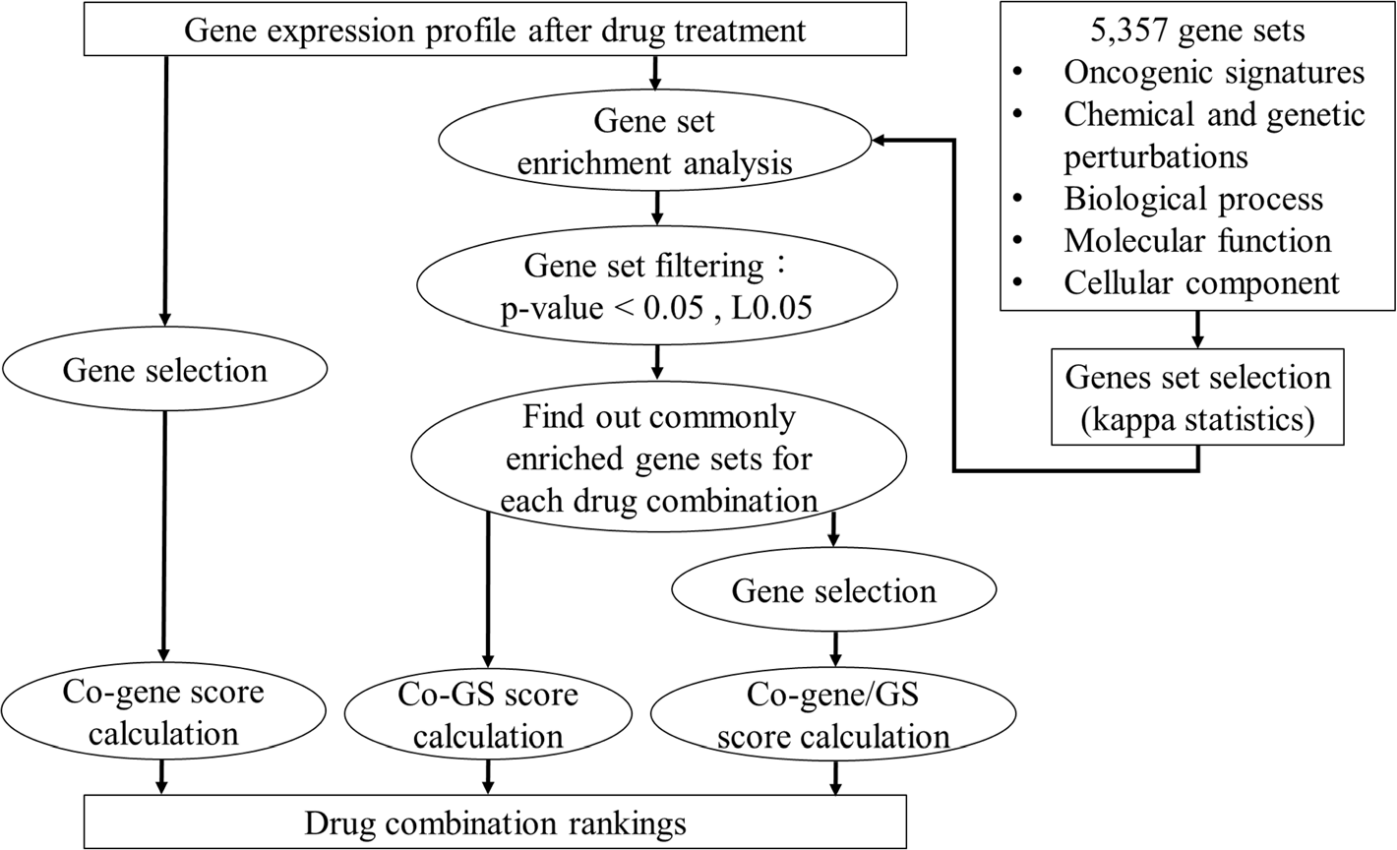
Flowchart of the three scoring methods. We devised three scores to rank the drug combinations in terms of synergy according to the gene expression profiles obtained from individual treatments. Two of the scores (co-gene and co-GS) were computed by the degree of overlap in disturbed genes or enriched gene sets between two drugs. Here activities and significance of changes in gene sets were modeled by a gene set enrichment analysis. For the co-gene/GS score of a drug pair, we computed an average percentage of overlapped genes across all commonly enriched gene sets. Drug pairs were ranked based on each of the prediction scores

Figure 2 is the flowchart of the proposed scoring methods. The three scoring methods were constructed based on the similarities of co-enriched genes/gene sets (representing biological functions and pathways) and/or gene components within the gene sets. Specifically, the co-GS and co-gene methods measure the degree of overlap between enriched gene sets and disturbed genes, respectively, between two drugs. On the other hand, the co-gene/GS score focuses on the similarity (intersection) of genes disturbed by drug treatments within the co-enriched gene sets. We ranked all drug combinations based on each prediction score; combinations with higher prediction scores are interpreted to have stronger synergistic effects, while low scores predict antagonistic effects. Mathematical definitions of the scores are provided in the Methods section. Since the gene sets represent biological functions, we were able to investigate the underlying mechanisms that account for the efficacy of drugs and the synergy of pairs of drugs.

### Performance evaluation of the proposed scores by DREAM gold standard

We assessed the performance of the proposed scores against the DREAM gold standard. Notably, all of the three scores achieved significant PC-index (empirical *P*-values < 0.01; Table 1), confirming our hypothesis that synergistic drugs perturb similar genes and biological functions, as well as similar genes related to a biological function. The intersection between genes disturbed by each drug (co-gene score) achieved a PC-index of 0.648 (*P*-value < 0.0001), outperforming all the 31 community-generated approaches in DREAM challenge (range, 0.613 to 0.420; mean, 0.510). At the gene set level, degree of overlapping in gene sets enriched in two drug treatments (co-GS) was predictive of synergistic effect (PC-index = 0.589 and *P*-value = 0.0036). Furthermore, integrating the gene set and gene levels, the co-gene/GS score achieved even higher performance (PC-index = 0.663 and *P*-value < 0.0001). Taken together, we inferred that reinforced regulation of similar biological functions is a crucial mechanism for drug synergy. The observation is consistent with previous findings that synergistic drugs affect the expression of genes involved in common pathways [15, 19, 20].

**Table 1:** Performance of prediction scores in the DREAM dataset

Scoring methods		PC-index	*P*-value
Co-gene score	$\frac{g_{d_1,{d}_2}}{G}$	0.648	<0.0001
Co-GS score	$\frac{n_{d_{1,}{d}_2}}{L}$	0.589	0.0036
Co-gene/GS score	$\frac{{\displaystyle {\sum}_l}\frac{N_{d_1,{d}_2}^l}{N^l}}{n_{d_{1,}{d}_2}}$	0.663	<0.0001

Notations: ${g}_{d_1,{d}_2}$, number of commonly regulated genes between drugs ${d}_1$ and ${d}_2$; $G$, total number of genes; ${n}_{d_{1,}{d}_2}$, number of gene sets with significant enrichment in both drugs; $L$, total number of gene sets; ${N}_{d_1,{d}_2}^l$, number of genes with significantly changes in both drug treatments within a co-enriched gene set *l*

### Further investigation into the co-gene/GS score

**Fig. 3:**
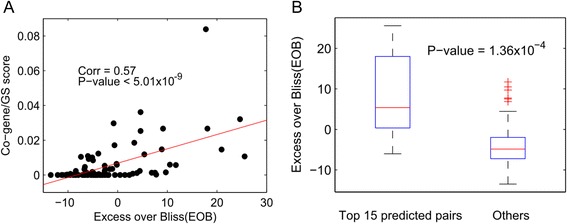
Evaluation of the co-gene/GS prediction score. **a** Scatter plot of the co-gene/GS prediction scores and excess over Bliss (EOB) values, which measures the experimentally assessed synergy of drugs. A significant positive correlation was identified between the two scores. **b** Box plots of EOB values between the top 15 predicted drug pairs and others. A significant rise in EOB was observed in the top pairs. The *P*-value was assessed by a one-tailed *t*-test

The co-gene/GS score was the best-performing method among our three scores and outperformed all prediction models proposed during the DREAM challenge. We further investigated its prediction power. The score was significantly positively correlated with the excess over Bliss (EOB) values, an experimentally derived indicator of synergy (*ρ* = 0.57 and *P*-value = 5.01 × 10^−9^; Fig. 3a). Table 2 tabulates the top 15 predicted synergistic drug pairs. These top pairs showed significantly higher EOB than the others (*t*-test one-tailed *P*-value = 1.36x10^−4^; Fig. 3b). Among them, the top synergistic drug pairs were camptothecin & mitomycin C (score = 0.084), camptothecin & doxorubicin (0.036), and H-7 & mitomycin C (0.032). Taken together, the proposed co-gene/GS score was strongly correlated with the EOB values and accurately predictive of synergistic effects of drug pairs.

**Table 2:** Top 15 synergistic drug pairs predicted by the co-gene/GS score in the DREAM dataset

Drug pair	Co-gene/GS score	Predicted rank	Gold-standard rank
Camptothecin & Mitomycin C	0.084	1	5
Camptothecin & Doxorubicin	0.036	2	16
H-7 & Mitomycin C	0.032	3	2
Methotrexate & Mitomycin C	0.030	4	28
Doxorubicin & Mitomycin C	0.027	5	4
Cycloheximide & H-7	0.027	6	9
Camptothecin & Etoposide	0.025	7	15
H-7 & Trichostatin A	0.019	8	19
H-7 & Rapamycin	0.017	9	27
Camptothecin & H-7	0.015	10	10
Etoposide & Mitomycin C	0.015	11	3
H-7 & Vincristine	0.012	12	43
H-7 & Monastrol	0.012	13	14
Cycloheximide & Methotrexate	0.011	14	64
Doxorubicin & H-7	0.011	15	1

**Fig. 4:**
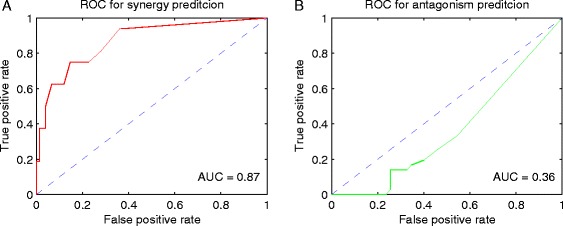
Receiver operating characteristic (ROC) curves for drug synergy and antagonism prediction. **a** ROC curve for drug synergy prediction. The area under the ROC curve (AUC) is 0.87. **b** ROC curve for drug antagonism prediction. The AUC is 0.36

We further analyzed the performance of the co-gene/GS score by AUC and precision analyses. The ROC curves for synergy and antagonism prediction are shown in Fig. 4a and b, respectively. The AUC for synergy prediction was 0.87, which outperformed all the methods proposed during the DREAM challenge (range, 0.813 to 0.207; mean, 0.515) and was even higher than the integrated method proposed in [13]. However, the AUC for antagonism prediction (AUC = 0.36) was worse than random guess; AUC of the 31 previous methods ranged from 0.677 to 0.337 with an average of 0.517. The precision for synergy and antagonism predictions was 62.5 and 47.2 %, respectively, higher than random guess (17.6 and 40.0 %) [13]. Notably, the high precision of our method in predicting drug synergy again outperformed all the methods proposed in the DREAM challenge.

Since the score was designed based on a gene set analysis scheme, the method also reports the commonly enriched biological functions for each drug combination. Take the top predicted pair as an example, camptothecin and mitomycin C were commonly enriched in gene sets of HOOI_ST7_TARGETS_UP, AMUNDSON_DNA_DAMAGE_RESPONSE_TP53, NIKOLSKY_BREAST_CANCER_19Q13.1_AMPLICON, KUMAMOTO_RESPONSE_TO_NUTLIN_3A_UP, and ZHU_SKIL_TARGETS_UP. Detailed lists of co-enriched gene sets for all drug combinations are provided in Additional file 1: Table S1.

### Application to the CMap dataset

After validating our method by the DREAM gold standard, we applied it to predict the synergy over a broader range of drugs analyzed in the CMap dataset. We used the expression profiles of MCF7 cell line treated with 1,118 compounds. A total of 2,588 instances (different compound-concentration combinations) were analyzed. Combinations of each drug pair at different concentrations were represented by the most synergistic one. The results of 1,038,710 drug combinations are shown in Additional file 2: Table S2. Top 10 synergistic pairs are listed in Table 3. These drug combinations are warranted candidates for further in vitro investigations.

**Table 3:** Top 10 predicted drug combinations in the CMap dataset

Drug combination	Co-gene/GS score	Rank
Prestwick-682 & MG-262	0.667	1
Cefazolin & Nocodazole	0.667	2
Anisomycin & Prednisolone	0.625	3
Lisinopril & Suramin sodium	0.600	4
Iopanoic acid & Butacaine	0.563	5
Alpha-ergocryptine & Clofazimine	0.529	6
Alpha-ergocryptine & Diloxanide	0.524	7
LM-1685 & Mepyramine	0.512	8
Genistein & Etoposide	0.504	9
Acetohexamide & Benzthiazide	0.500	10

## Discussion

In the proposed study we designed three prediction models to fully test the hypothesis that synergy of drugs can be achieved by targeting common biological pathways or genes. The hypothesis was confirmed by using the DREAM in vitro dataset. Specifically, the co-gene/GS scoring system, measuring commonly gene-level disturbances within co-enriched gene sets between two drugs achieved high prediction accuracy, AUC, and precision for drug synergy and outperformed all proposed methods in the DREAM challenge.

Another advantage of our method is the ability to identify the underlying functions/mechanisms that lead to the efficacy of drug treatments and the synergy of drug combinations. For example, for the top synergistic drug pair reported in the DREAM gold standard, doxorubicin & H-7, we identified 14 commonly enriched gene sets (Table 4). One of the gene sets, “NEWMAN_ERCC6_TARGETS_DN”, consists of genes involved in DNA repair- and transcription-related pathways [21]. Both doxorubicin and H-7 are known to affect transcription in cells. Doxorubicin is widely used as a first-choice anticancer drug in a variety of tumors, including breast, lung, ovary, thyroid, and leukemia. It intercalates with DNA base pairs, binds to topoisomerase II, and causes DNA damages that may activate apoptotic pathways when the attempt to repair DNA breaks fails [22, 23]. In addition, doxorubicin has also been reported to directly affect DNA transcription via inducing histone eviction in promoter regions [24]. H-7 is well-characterized for its ability to deregulate DNA transcription. It acts as a protein kinase C (PKC) inhibitor [25]. PKC family has been reported to affect the activity of some transcription factors and regulate gene transcription [26, 27]. As an inhibitor of PKC, treatment of H-7 affects the downstream pathways of PKC and thereby alters the transcription processes of cell. Taken together, both these two drugs can change transcription activity, which is consistent with our findings that the treatments of these two drugs co-regulate the gene set, “NEWMAN_ERCC6_TARGETS_DN”, composed of transcription-related genes.

**Table 4:** Commonly enriched gene sets of doxorubicin and H-7 in the DREAM dataset

Gene set	MSigDB category
NEWMAN_ERCC6_TARGETS_DN	Chemical and genetic perturbations
LEE_NEURAL_CREST_STEM_CELL_DN	Chemical and genetic perturbations
ODONNELL_METASTASIS_UP	Chemical and genetic perturbations
CERVERA_SDHB_TARGETS_2	Chemical and genetic perturbations
OSADA_ASCL1_TARGETS_UP	Chemical and genetic perturbations
YAUCH_HEDGEHOG_SIGNALING_PARACRINE_UP	Chemical and genetic perturbations
HINATA_NFKB_TARGETS_KERATINOCYTE_DN	Chemical and genetic perturbations
TAVAZOIE_METASTASIS	Chemical and genetic perturbations
VALK_AML_CLUSTER_7	Chemical and genetic perturbations
MIKKELSEN_NPC_ICP_WITH_H3K27ME3	Chemical and genetic perturbations
HOELZEL_NF1_TARGETS_UP	Chemical and genetic perturbations
PTEN_DN.V2_UP	Oncogenic signatures
KRAS.DF.V1_DN	Oncogenic signatures
Response to wounding	Gene ontology terms

Applying the prediction model to a huge collection of drug-treatment profiles deposited in CMap, we successfully identified potential synergistic drug combinations (Table 3). Taking the ninth pair, genistein & etoposide, as an example, the two drugs showed a similar enrichment in the gene set “peptide transporter activity” (Table 5), indicating their co-regulation of transporters such as *MRP1* (*ABCC1*), an drug-efflux pump causing cancer cell to attain resistance to some antitumor drugs [28]. Genistein, a natural isoflavonoid compound appearing in citrus fruits and soybean, has been reported to have antitumor effect on several types of cancers, including breast cancer [29]. It is also capable of inhibiting the multidrug resistance resulting from the well-known drug efflux proteins, such as *MRP1*, P-glycoprotein, and other ABC (ATP-binding cassette) proteins [30–33]. Etoposide, on the other hand, is a topoisomerase II inhibitor and has been reported to induce apoptosis in MCF7 cell line [34]. Being a substrate of *MRP1*, P-glycoprotein and other multidrug resistance-associated proteins, etoposide is ineffective to suppress tumor growth when these proteins are largely produced [35, 36]. Previous in vitro studies showed that genetic deletion of the MRP proteins greatly restores the sensitivity of tumor cells to etoposide due to the minor contributions of other drug-efflux proteins, such as P-glycoprotein, to the drug resistance of etoposide [36, 37]. Taken together, when these two drugs are combined, the inhibitory effect on multidrug resistance of genistein may sensitize cellular responses to etoposide, thus intensifying the antitumor effects through apoptosis.

**Table 5:** Commonly enriched gene sets of genistein and etoposide in the CMap dataset

Gene set	MSigDB category
DE_YY1_TARGETS_DN	Chemical and genetic perturbations
Peptide transporter activity	Gene ontology terms
TAP1 binding	Gene ontology terms

There are some limitations in our methods. First, the gene set enrichment score was calculated by summing all the expression values, thus the topological relationships between genes were not considered. This may lead to an underestimation of those upstream genes that are able to cause significant disturbance pathway-wise even when they are slightly changed. Future studies may extend this work by using other gene set scoring methods that weight content genes based on topological information [38, 39] and significance of changes [16, 40]. Besides, this scoring scheme ignores the directional (i.e., upward and downward) changes in genes of a gene set. This may cause biases in estimating the influence of expressional changes on biological functions. Thirdly, in the calculation of prediction scores we considered a simple intersection, but ignored the directional agreement of changes in two gene sets or two genes. Further improvement to our method that tackles this concern may provide better biological insights. Fourthly, our proposed similarity-based scoring methods were highly predictive of drug synergy, but did not performed well in predicting drug antagonism. This was consistent to the findings from the DREAM challenge: hypotheses needed to predict synergy and antagonism may be quite different [13]. Furthermore, drug pairs could attain synergistic/antagonistic effects by varied mechanisms. Our proposed method, even though proved to have high prediction power, may only address one specific type of mechanisms. To fully explore all possibilities and further improve the robustness of the prediction method, future studies may integrate diverse data types of the treated cells, such as methylation and gene mutation data, and combine different prediction models, as well as other hypothesis.

## Conclusions

In this paper, we comprehensively tested and confirmed the hypothesis that synergy of drugs can be achieved by regulating common biological functions and genes. A synergy-predicting score was proposed and validated by an experimentally assessed gold standard. The prediction performance of this simple score was better than previous methods. We applied our devised method to a larger collection to screen for potential drug combinations. We also demonstrated that the method not only achieves accurate prediction but also investigates the underlying mechanisms of drug synergy.

## Additional files

Additional file 1: Table S1. List of co-enriched gene sets for all drug combinations in the DREAM dataset. (XLSX 35 kb) Additional file 2: Table S2. Prediction of all 1,038,710 drug combinations in the CMap dataset. (XLSX 28927 kb)
